# DNA Repair Gene XRCC1 and XPD Polymorphisms and Gastric Cancer Risk: A Case-Control Study Outcome from Kashmir, India

**DOI:** 10.1155/2018/3806514

**Published:** 2018-08-26

**Authors:** Bushra Nissar, Showkat A. Kadla, Nuzhat Shaheen Khan, Idrees A. Shah, Misbah Majid, Falaque ul Afshan, Bashir Ahmad Ganai

**Affiliations:** ^1^Department of Biochemistry, University of Kashmir, Srinagar, India; ^2^Department of Gastroenterology, Government Medical College and Hospital, Srinagar, India; ^3^Department of MRDG, Indian Institute of Science, Bangalore, India

## Abstract

Coding polymorphisms in several DNA repair genes have been reported to affect the DNA repair capacity and are associated with genetic susceptibility to many human cancers, including gastric cancer. An understanding of these DNA repair gene polymorphisms might assess not only the risk of humans exposed to environmental carcinogens but also their responses to different therapeutical approaches, which target the DNA repair pathway. In the present study, polymorphic variants of two DNA repair genes, XRCC1 Arg399Gln and XPD Lys751Gln, were chosen to be studied in association with gastric cancer susceptibility in the Kashmiri population. A total of 180 confirmed cases of gastric cancer (GC) and 200 hospital-based controls from Government Shri Maharaja Hari Singh Hospital, Srinagar, were included in the study. The genotyping for XRCC1 and XPD genes was carried out by polymerase chain reaction-restriction fragment length polymorphism. We found that tobacco smoking is strongly associated with GC risk (OR = 25.65; 95% CI: 5.49–119.7). However, we did not find any association of polymorphism of XRCC1 Arg399Gln (OR = 1.56; 95% CI: 0.32–7.82) and XPD Lys751Gln (OR = 0.46; CI: 0.10–2.19) with GC risk in the study population. The combination of genotypes and gender stratification of XRCC1 and XPD genotypic frequency did not change the results. Consumption of large volumes of salt tea was also not associated with gastric cancer risk. Polymorphic variants of XRCC1 Arg399Gln and XPD Lys751Gln are not associated with the risk of gastric cancer in the Kashmiri population. However, replicative studies with larger sample size are needed to substantiate the findings.

## 1. Introduction

Gastric cancer is the fifth most common cancer as regards the prevalence but has the third highest incidence of deaths worldwide [1]. In India, gastric cancer is the third most common cancer in males [2]. However, in the Kashmir Valley, GC is the most frequently encountered cancer in males and ranks third in females [3]. Kashmir, with its unique dietary practices and a different geographical location, is reportedly exposed to a particular set of environmental and dietary carcinogens conceding a unique pattern of cancer distribution. It is a high-incidence area burdened with most commonly occurring cancers, in particular the cancers of the gastrointestinal tract [4]. The Kashmiri population is unceasingly being exposed to mutagenic and carcinogenic aromatic amines, nitrates, and N-nitroso compounds through the environmental factors and/or the local food stuffs and other sources that can form DNA adducts in vivo and lead to DNA damages, which eventually lead to tumorigenesis [5].

The human genomic integrity is, however, maintained by the DNA repair systems. Multiple DNA repair pathways exist to provide a distinct but overlapping protection against the DNA-damaging exposures. There are at least 4 different pathways of DNA repair that function on specific types of DNA damages including base excision repair (BER), nucleotide excision repair (NER), double-stranded break repair (DSBR), and mismatch repair (MMR) [6, 7]. Widely cited evidences suggest that the base excision repair (BER) and NER are the most versatile mechanisms of DNA repair. The BER is a DNA repair mechanism that repairs the small aberrations such as oxidized or reduced bases, fragmented or nonbulky adducts, or those produced by methylating agents [8]. X-ray repair cross-complementary 1 (XRCC1) is the key enzyme of the BER pathway [9, 10]. The XRCC1 gene maps to the chromosome 19q13.2 and consists of 17 exons. XRCC1 has no enzymatic activity and acts as a scaffold and the modulator of different activities involved in the BER by interacting with and bringing together poly ADP-ribose polymerase (PARP), DNA ligase III, and DNA pol *β* via its various domains and polynucleotide kinase (PNK) at the site of DNA damage [11, 12]. Thus, the XRCC1 protein provides a physical link between the incision and sealing steps of the BER pathway. It is a multidomain protein which interacts with the nicked as well as the gapped DNA associated with the DNA pol *β*, suggesting that this protein might be independently involved in the DNA damage recognition [13].

Although more than 300 single-nucleotide polymorphisms (SNPs) in the XRCC1 gene have been reported, three SNPs are common, which lead to the amino acid substitution in XRCC1 codon 194 (rs1799782), 280 (rs25489), and 399 (rs25487). These nonconservative amino acid changes may alter the XRCC1 function and increase the chances of DNA damage. Out of the three polymorphisms, the Arg399Gln polymorphism resulting from a G to A nucleotide substitution occurs in the PARP binding domain of the XRCC1 protein which may affect the complex assembly and/or the repair efficiency [14].

NER is another versatile DNA damage removal pathway that eliminates a wide variety of damages to the human genome including UV-induced photoproducts, bulky monoadducts, cross-links, and oxidative damage [15]. One of the major proteins of the NER pathway is xeroderma pigmentosum D (XPD), which is also known as excision-repair cross-complementing complementation group 2 (ERCC2). The XPD gene maps to the chromosome 19q13.3 and is composed of 23 exons. XPD is an ATP-dependent helicase within the multisubunit, TFIIH, and transcription repair factor II helicase complex, which participates in the DNA unwinding during NER and basal transcription [16]. As a part of the TFIIH transcription factor/complex, amino acid variants in the different domains of XPD may alter the different protein interactions and result in expression of different phenotypes [17, 18]. Several SNPs have been identified in the XPD gene, of which Lys751Gln (rs13181) is one of the commonly found SNPs that results in amino acid change [14]. The XPD 751 polymorphism modifies the amino acid electronic configuration in a domain that is important for the interaction with helicase activator p44 and may produce the most relevant change in the XPD function [19].

The DNA repair capacity of the individuals varies as a result of inheritance, environmental factors, and physiological factors [20]. There are a number of studies suggesting that the polymorphisms in the DNA repair genes may contribute to the susceptibility to cancers through an alteration of repair capacity [21]. Among the known genetic polymorphisms of the DNA repair genes, XRCC1 and XPD have been most commonly studied [8]. The previous findings regarding the association of these polymorphisms and the risk of gastric cancer have not been entirely consistent. A number of evidences have documented the existence of an association of XRCC1 and XPD with an increased risk of gastric cancer [22–26]. However, an analysis of the same variants resulted in lack of any association in other population-based studies [27–30]. Therefore, the present case-control study was carried out to investigate whether the polymorphisms in the DNA repair genes XRCC1 (Arg399Gln) and XPD (Lys751Gln) modulate the risk of developing GC in Kashmir, which has a relatively high incidence of GC.

## 2. Subjects and Methods

### 2.1. Study Subjects and Data Collection

A total of 180 histologically confirmed GC patients and 200 age- and gender-matched control subjects were recruited from Government Shri Maharaja Hari Singh (SMHS) Hospital, Srinagar. Only those subjects who had no previous history of cancer and were admitted for minor ailments like diarrhoea and hernia or showed up for ophthalmological and dental check-ups were recruited as controls. The data on the gastric cancer patients was obtained by conducting personal interviews of all the subjects and from their medical records. Structured questionnaires were used to collect the information regarding the age, sex, place of residence, smoking status, family history, and other confounding factors of interest. All the patients duly signed the informed consent, and the recruitment was initiated following the approval from the ethics committee. The criteria for including a subject as a case in the study were patients having histopathologically and endoscopically proven case of gastric cancer, patients willing to participate in the study, and patients belonging to the Kashmir region only.

Under the following conditions, the patients were excluded from the study: patients who have gastric cancer simultaneously suffering from any other malignancy, patients who had received prior chemotherapy or radiotherapy for gastric cancer, and patients who refused to participate in the study. The healthy controls were matched to the cases by age, gender, and region and were recruited from the various in-patient wards of SKIMS. The inclusion criteria for control samples were patients asymptomatic to gastric cancer, patients without a history of cancer, and residents of the Kashmir region. And the exclusion criteria were patients suffering from any kind of malignancy and patients suffering from any disease that affected their life style and/or dietary habits like diabetes. 2–5 ml of blood sample was collected from each subject in EDTA-coated vials properly labelled and stored in 80°C prior to use. Genomic DNA was isolated from the whole blood samples of all the subjects manually using the standard phenol/chloroform method [31].

### 2.2. Genotyping of XRCC1 Codon 399

**Figure 1:**
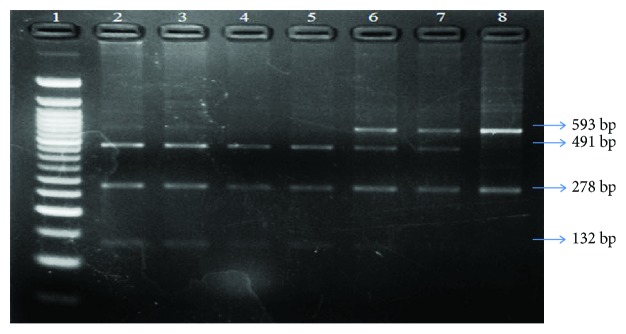
Representative gel picture showing *Msp*I-digested amplicons of XRCC1 codon 399 run on 3% agarose gel. Lane 1: 50 bp DNA ladder. Lanes 2, 3, 4, and 5: 132 bp, 278 bp, and 461 bp Arg/Arg genotype. Lanes 6 and 7: 132 bp, 278 bp, 461 bp, and 593 bp Arg/Gln genotype. Lane 8: 278 bp and 593 bp Gln/Gln genotype.

The XRCC1 genotypes (Arg399Gln/G to A) were determined by the polymerase chain reaction (PCR) amplification and restriction digestion with *Msp*I (Thermo Fisher Scientific Inc. (EU), Lithuania). A region of 871 bp carrying the restriction site for *Msp*I was amplified by PCR using the following set of primers: forward primer (5′-CAG TGG TGC TAA CCT AAT C-3′) and reverse primer (5′- AGT AGT CTG CTG GCT CTG G-3′) (Integrated DNA Technologies, Coralville, Iowa). All the reactions were carried out in a total reaction volume of 25 *μ*l containing 50–75 ng of genomic DNA template and 1 U of Taq polymerase (Thermo Fisher Scientific Inc. (EU), Lithuania). The PCR conditions were initial denaturation for 10 minutes at 94°C, followed by 30 cycles each of 1-minute denaturation at 94°C, 30-second annealing phase at 62°C, and 45-second extension at 72°C, followed by a final extension at 72°C for 7 minutes. The PCR amplicon (871 bp) was digested overnight at 37°C with *Msp*I. The restriction/digestion products were resolved on 3% agarose gel containing 0.5 *μ*g/ml ethidium bromide using a gel electrophoresis system at 100 V for 30–40 min and visualized under UV light. The *Msp*I digestion resulted in three fragments of 461 bp, 278 bp, and 132 bp for homozygous wild genotype (Arg/Arg), two fragments of 593 bp and 278 bp for homozygous variant genotype (Gln/Gln), and four fragments of 593 bp, 461 bp, 278 bp, and 132 bp for heterozygous genotype (Arg/Gln) (Figure 1).

### 2.3. Genotyping of XPD Codon 751

**Figure 2:**
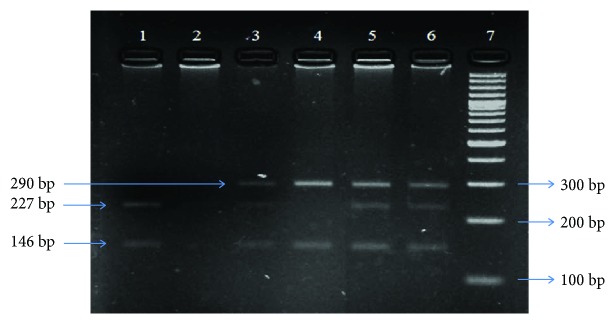
Representative gel showing *Pst*I-digested amplicons of XPD codon 751 run on 3% agarose gel. Lane 7: 100 bp DNA ladder. Lane 4: 146 bp and 290 bp Lys/Lys genotype. Lanes 3, 5, and 6: 146 bp, 227 bp, 290 bp, and 63 bp Lys/Gln genotype. Lanes 1 and 2: 146 bp, 227 bp, and 63 bp Gln/Gln genotype.

The XPD genotypes (Lys751Gln/A to C) were determined by the PCR amplification and restriction digestion of the products with *Pst*I (Thermo Fisher Scientific Inc. (EU), Lithuania). A region of 436 bp carrying the restriction site for *Pst*I was amplified by PCR using the following set of primers: forward primer (5′- GCC CGC TCT GGA TTA TAC G-3′) and reverse primer (5′- CTA TCA TCT CCT GGC CCC C-3′) (Integrated DNA Technologies, Coralville, Iowa). All the reactions were carried out in a total reaction volume of 25 *μ*l containing 50–75 ng of genomic DNA template and 1 U of Taq polymerase (Thermo Fisher Scientific Inc. (EU), Lithuania). The PCR conditions were initial denaturation for 10 minutes at 94°C, followed by 30 cycles each of 1-minute denaturation at 94°C, 30-second annealing phase at 65°C, and 45-second extension at 72°C, followed by a final extension at 72°C for 7 minutes. The amplified product was resolved on 2% agarose gel containing 0.5 *μ*g/ml ethidium bromide and visualized under UV light using a transilluminator system. The PCR amplicon (436 bp) was digested overnight at 37°C with *Pst*I. The restriction/digestion products were resolved on 3% agarose gel containing 0.5 *μ*g/ml ethidium bromide using a gel electrophoresis system at 100 V for 30–40 min and visualized under UV light. The *Pst*I digestion resulted in two fragments of 290 bp and 146 bp for homozygous wild genotype (Lys/Lys), three fragments of 227 bp, 146 bp, and 63 bp for homozygous variant genotype (Gln/Gln), and four fragments of 290 bp, 227 bp, 146 bp, and 63 bp for heterozygous genotype (Lys/Gln) (Figure 2).

### 2.4. Statistical Analysis

Numbers and percentages were calculated and presented for categorical variables as well as means and standard deviations (SD). Subjects were stratified into various groups based on gender and various possible genotype combinations. Conditional logistic regression models were used to calculate odds ratios (ORs) and their corresponding 95% confidence intervals (CIs) to assess the association of the genotypes with gastric cancer risk and to assess the possible gene-gene interactions. Adjustment was made with known risk factors like residence, gender, family history, chillies, exposure to X-ray, smoking, and dried foods. All statistical analyses were done using Stata software, version 12 (STATA Corp., College Station, TX, USA). Two-sided *P* values < 0.05 were considered statistically significant.

## 3. Results

The present study included a total of 380 subjects including 180 cases and 200 matched controls. The distribution of demographic and clinicopathological characteristics of all the subjects is given in Table 1. The mean age ± SD of the cases was 61.29 ± 11.06, and the female-to-male ratio turned out to be 3.28. More number of cases (80.35%) resided in the rural areas than the respective controls. Smoking habit was prevalent in cases (61.21%) than in controls. However, we did not find any significant differences in the X-ray exposure and consumption of various dietary items like dried foods and chillies among cases versus controls (*P* value > 0.05). We did not find any significant difference in the minor allele frequencies of XRCC1 Arg399Gln and XPD Lys751Gln between cases and controls (Table 1).

Table 2 represents the association of various important risk factors with GC in the Kashmiri population. Compared to the subjects who drank ≤3 cups (1 cup ≈ 200 ml) of salt tea, we did not find any significant association of consumption of larger volumes of salt tea with gastric cancer in Kashmir (*P* for trend = 0.937). An indication of high risk of gastric cancer in the subjects who had a positive family history of cancer was observed; however, it did not achieve statistical significance. In the present study, compared to never smokers, tobacco smoking was strongly associated with gastric cancer in Kashmir (OR = 25.65; 95% CI: 5.49–119.7) Table 2.

The genotypic frequencies of XRCC399 and XPD751 genotypes among cases and controls are represented in Table 3. We did not find any significant association of studied polymorphisms of XRCC1 (OR = 1.56; 95% CI: 0.32–7.82) and XPD (OR = 0.46; CI: 0.10–2.19) with gastric cancer in the study population. In order to test whether the individual polymorphisms in the two DNA repair genes might interact and modify the risk of developing gastric cancer, ORs and 95% CIs were estimated for the combined genotypes of XRCC1 Arg399Gln and XPD Lys751Gln polymorphisms; however, no effect modifications were observed by these polymorphisms in the current study (*P* interaction = 0.793) Table 3. On further stratification of the subjects on the basis of gender, we did not find any significant association of XRCC1 Arg399Gln and XPD Lys751Gln polymorphisms with gastric cancer (Supplementary Table 1).

## 4. Discussion

In the current study, we studied whether the polymorphisms in the two DNA repair genes, XRCC1 and XPD, involved in BER and NER pathways, respectively, are implicated in the development of gastric cancer in the Kashmiri population. Our results showed smoking as an independent risk factor for gastric cancer in Kashmir; however, no evidence of any association between the polymorphisms XRCC1 Arg399Gln and XPD Lys751Gln and the risk of GC was found in this population.

The DNA repair pathways are responsible for maintaining the genomic integrity [8, 32, 33] and protection against carcinogenesis [34], through the reversal of the DNA damages [35]. The inherited functional polymorphisms or an accumulation of the mutations in the DNA repair genes may, however, influence the hosts' DNA repair capacity and thus modulate the risk of carcinogenesis [8]. A growing number of evidences suggest that the single-nucleotide polymorphisms (SNPs) of the common DNA repair genes are associated with several sporadic cancers [36, 37], including GC [8, 38].

Rare variants with minor allele frequency less than 1% make a contribution to the genetic risk of various complex diseases [39]. Of the various genetic variants, single-nucleotide polymorphisms (SNPs) are known to play a crucial role in the progression of cancer. Although many SNPs have been reported to be associated with the risk of various cancers, the variants identified so far explain only a very small part of the cases, suggesting that many more genetic determinants exist, which are yet to be characterized [40].

In the present study, we detected the polymorphisms of XRCC1 and XPD genes in 180 gastric cancer cases and 200 matched healthy controls. The results showed no statistically significant differences in the allelic or in the genotypic frequencies of XRCC1 Arg399Gln and XPD Lys751Gln polymorphisms between the control group and the patients with GC. The combined effects of studied polymorphism of the two genes also did not show any increase in the risk of GC. Our results seem to be in agreement with previous studies that suggested an insignificant association of XRCC1 Arg399Gln genotypes with GC risk in Korean [41], Chinese [27], Polish [42], and Brazilian populations [28]. Our results for XPD Lys751Gln polymorphism are also in agreement with the results from previous studies in Polish [42], Turkish [43, 44], Italian [29], and Chinese populations [45]. However, unlike our results, some earlier studies have reported a significant association of XRCC1 Arg399Gln and XPD Lys751Gln polymorphism [22–26] with the risk of GC. The main reason for this inconsistence in findings could be the difference in ethnicities and study design and smaller sample size.

Among the various parameters, tobacco smoking status showed an increased risk of gastric cancer in smokers of our population (*P* value < 0.05, OR = 25.65; 95% CI = 5.49–119.75), albeit wider CIs due to a low number in the model. The possible reason for this increased risk could be that smokers are exposed to a large number of carcinogens [46–48]. Tobacco smokers are exposed to many toxic compounds such as nicotine, nitric oxide, carbon monoxide, polyaromatic hydrocarbons (PAH), nitrosamines, and aromatic amines [49–53]. Tobacco smoke induces the development of precursor lesions including gastritis, ulceration, and intestinal metaplasia, which ultimately lead to GC [54]. The results from the present study are in agreement with several case-control and cohort studies that reported an association of tobacco smoking and GC risk [55–57]. An earlier study from the same population has reported tobacco smoking to be associated with esophageal squamous cell carcinoma (ESCC) as well [48].

The consumption of hot salted alkaline tea (noon chai) is considered one of the factors contributing to gastric cancer risk in Kashmir [58]. In Kashmir, salt tea is brewed in a unique manner, usually with the addition of sodium bicarbonate, which makes the tea alkaline. The frequent consumption of salt tea results in an exceptionally high exposure to carcinogenic amines including methylamines, dimethylamine, pyrrolidine, and methylbenzylamine, besides the presence of preformed N-nitrosamines including N-nitrosodimethylamine (NDMA), N-nitrosoproline (NPRO), and N-nitrosopipecolic acid (NPIC), along with three nonvolatile N-nitroso compounds (yet to be identified), formed during the preparation of salt tea via traditional methods [47, 59]. Thus, the N-nitroso compounds and their possible endogenous formation due to an increased consumption of salt tea may be a critical factor for the high occurrence of gastric cancer in Kashmir [5, 47, 59, 60]. Furthermore, an earlier study in the Kashmiri population also reported a significant association between consumption of large volumes of salt tea and ESCC [61]. However, in our study, we did not find any significant association of consumption of large volumes of salt tea with gastric cancer risk, which can be attributed to a small sample size and needs further validation.

Though adjustments of the results for multiple potential confounding factors are the major strengths of this study, but our study was based on the modest sample size to detect any of the possible genetic associations and gene-environment interactions. The current sample size was also found to be enough for us to attain the 80% power for this study. Although a limited staff interviewed the subjects, similar to other case-control studies with retrospective exposure assessments, recall and interviewer bias may also be a concern in this study but is unlikely to affect the outcome of the study. In conclusion, our study reports that the polymorphisms of XRCC1 Arg399Gln and XPD Lys751Gln were not associated with the GC risk in the Kashmiri population. However, the factors like smoking may contribute to an increased risk of this cancer. Further replicative studies with larger sample size are warranted to validate our findings.

## Figures and Tables

**Table 1:** Demographic characteristic of gastric cancer cases and controls.

Characteristics	Cases *n* (%)	Controls *n* (%)	*P* value^1^
Total	180 (100)	200 (100)	—
Age			
>50	33 (18.33)	34 (17.0)	—
≤50	147 (81.67)	166 (83.0)	
Gender			
Male	138 (76.67)	155 (77.50)	—
Female	42 (23.33)	45 (22.50)	
Residence			
Urban	34 (19.65)	56 (28.43)	0.050
Rural	139 (80.35)	141 (71.57)	
Smoking			
Never	64 (38.79)	172 (89.12)	0.000
Ever	101 (61.21)	21 (10.88)	
Family history			
No	164 (94.80)	185 (95.85)	0.631
Yes	9 (5.20)	8 (4.15)	
Dried foods			
No	1 (0.61)	0 (0.00)	0.323
Yes	164 (99.39)	161 (100)	
X-rays			
No	92 (59.74)	74 (51.75)	0.166
Yes	62 (40.26)	69 (48.25)	
Chillies			
Low	32 (18.50)	39 (24.38)	0.108
Moderate	57 (32.95)	37 (23.13)	
High	84 (48.55)	84 (52.50)	
Allele type			
XRCC1			
G	204 (56)	218 (54.5)	0.48
A	156 (43)	182 (45.5)	
XPD			
A	263 (73.05)	283 (70.75)	0.54
C	97 (26.94)	117 (29.25)	

*n* = number of individuals. ^1^Chi-square test (*χ* ^2^) was used to calculate *P* values for categorical variables.

**Table 2:** Association of various dietary and environmental factors with gastric cancer in Kashmir.

Number of cups of salt tea	Cases *n* (%)	Controls *n* (%)	Unadjusted OR (95% CI)	Adjusted^1^ OR (95% CI)	*P* value
1 to 3 cups	47 (27.49)	58 (39.19)	Referent	Referent	—
4 to 6 cups	108 (63.16)	83 (56.08)	1.46 (0.86–2.49)	1.14 (0.32–3.99)	
Above 6 cups	16 (9.36)	7 (4.73)	2.04 (0.70–5.96)	0.82 (0.11–5.97)	
P trend			0.100	0.937	
Family history					
No	164 (94.80)	185 (95.85)	Referent	Referent	0.631
Yes	9 (5.20)	8 (4.15)	1.40 (0.52–3.79)	4.80 (0.55–41.9)	
Smoking status					
Never	64 (38.79)	172 (89.12)	Referent	Referent	<0.001
Ever	101 (61.21)	21 (10.88)	29.38 (9.28–93.05)	25.65 (5.49–119.7)	

OR = odds ratio; CI = confidence interval. ^1^Adjusted ORs (95% CIs) were obtained in conditional logistic regression models with adjustment for residence, gender, family history, chillies, X-ray, smoking, and dried foods. Numbers may not add up to the total due to missing values in some variables.

**Table 3:** Independent and combined genotypic distribution of XPD and XRCC1 in gastric cancer cases and matched controls.

Genotypes	Cases *n* (%)	Controls *n* (%)	Unadjusted OR^1^ (95% CI)	Adjusted^10^ OR (95% CI^2^)
Total	180 (100)	200 (100)	—	—
XRCC1 399				
Arg/Arg^3^	56 (31.11)	47 (23.50)	Referent	Referent
Arg/Gln^4^	92 (51.11)	124 (62.00)	0.65 (0.41–1.03)	0.95 (0.32–2.84)
Gln/Gln^5^	32 (17.78)	29 (14.50)	0.99 (0.52–1.88)	1.56 (0.32–7.82)
Arg/Gln + Gln/Gln	124 (68.89)	153 (76.50)	0.71 (0.46–1.11)	1.01 (0.34–3.01)
XPD 751				
Lys/Lys^6^	107 (59.44)	114 (57.00)	Referent	Referent
Lys/Gln^7^	49 (27.22)	55 (27.50)	0.90 (0.57–1.41)	1.40 (0.45–4.32)
Gln/Gln^8^	24 (13.33)	31 (15.50)	0.82 (0.43–1.53)	0.46 (0.10–2.19)
Lys/Gln + Gln/Gln	73 (40.56)	86 (43.0)	0.87 (0.59–1.29)	0.95 (0.40–2.27)
Combined genotypes^9^ (*P* interaction = 0.793 and SE = 0.568)				
XRCC1 Arg/Arg + XPD Lys/Lys	32 (17.78)	25 (12.50)	Referent	Referent
XRCC1 Arg/Arg + XPD (Lys/Gln + Gln/Gln)	24 (13.33)	22 (11.00)	0.79 (0.35–1.74)	2.72 (0.28–26.78)
XRCC1 (Arg/Gln + Gln/Gln) + XPD Lys/Lys	75 (41.67)	89 (44.50)	0.67 (0.35–1.26)	2.09 (0.32–13.61)
XRCC1 (Arg/Gln + Gln/Gln) + XPD (Lys/Gln + Gln/Gln)	49 (27.22)	64 (32.00)	0.60 (0.32–1.12)	1.31 (0.25–6.82)

^1^OR = odds ratio. ORs (95% CIs) were obtained from conditional logistic regression models. ^2^Confidence interval. ^3^XRCC1 wild genotype. ^4^XRCC1 heterozygous genotype. ^5^XRCC1 mutant genotype. ^6^XPD wild genotype. ^7^XPD heterozygous genotype. ^8^XPD mutant genotype. ^9^Genotype effects of both genes. ^10^Adjusted ORs (95% CIs) were obtained in conditional logistic regression models with adjustment for residence, gender, family history, chillies, X-ray, smoking, and dried foods.

## Data Availability

The data used to support the findings of this study are available from the corresponding author upon request.

## Abbreviations

BER: Base excision repair
NER: Nucleotide excision repair
XRCC1: X-ray repair cross-complementary 1
XPD: Xeroderma pigmentosum D.

## Supplementary Materials

Supplementary Materials Supplementary Table 1: odds ratio and 95% CI of XRCC1 and XPD genotypes in gastric cancer cases and controls stratified by gender.
