# Supplementary material for: DNA Repair Gene XRCC1 and XPD Polymorphisms and Gastric Cancer Risk: A Case-Control Study Outcome from Kashmir, India
Source: Anal Cell Pathol (Amst). 2018 Aug 26;2018:3806514. doi: 10.1155/2018/3806514 (PMC6129361; doi:10.1155/2018/3806514)
Supplement: Supplementary Materials — Supplementary Table 1: odds ratio and 95% CI of XRCC1 and XPD genotypes in gastric cancer cases and controls stratified by gender. [file 3806514.f1.pdf]

Supplementary table 1: Odds Ratio and 95% C.I of XRCC1 and XPD genotypes in gastric cancer cases and controls stratified by gender.

Variable	XRCC1				XPD			
	Cases (%)	Controls (%)	Unadjusted OR (95% CI)	Adjusted[1] OR (95 % CI)	Cases (%)	Controls (%)	Unadjusted OR (95% CI)	Adjusted OR (95 % CI)
**Wild+ male**	44 (31.88)	43 (27.74)	Referent	Referent	79 (57.25)	90 (58.06)	Referent	Referent
**Hetro+ male**	70 (50.72)	92 (59.35)	0.78 (0.47 – 1.31)	2.18 (0.50 – 9.58	42 (30.43)	43 (27.74)	1.01 (0.62 – 1.65)	2.04 (0.44 – 9.40)
**Mutant+ male**	24 (17.39)	20 (12.90)	1.25 (0.60 – 2.60)	6.68 (0.40 – 111.3)	17 (12.32)	22 (14.19)	0.86 (0.41 – 1.82)	2.18 (0.47 – 100.6)
**Wild+ female**	12 (28.57)	4 (8.89)	Referent	Referent	28 (66.67)	24 (53.33)	Referent	Referent
**Hetro+ female**	22 (52.38)	32 (71.11)	0.29 (0.09 – 0.98)	0.56 (0.04 – 8.08)	7 (16.67)	12 (26.67)	0.52 (0.17 – 1.57)	1.06 (0.10 – 11.18)
**Mutant+ female**	8 (19.05)	9 (20.00)	0.39 (0.09 – 1.69)	0.21 (0.01 – 6.89)	7 (16.67)	9 (20.00)	0.70 (0.21 – 2.28)	0.17 –(0.01 – 3.57

Foot notes supplementary table 1
OR=odds ratio. CI = confidence Intervals
[1] Adjusted ORs (95 % CIs) was obtained in conditional logistic regression models adjusted for residence, gender, family history, chillies, X-ray, smoking and dried foods.
